# German rehabilitation after total hip or knee arthroplasty through Dutch eyes: a qualitative focus group pilot study

**DOI:** 10.1186/s13104-026-07763-0

**Published:** 2026-03-13

**Authors:** Yvet Mooiweer, Gesine H. Seeber, Manna Alma, Annemiek Kuipers, Mike Keasberry, Sophie Thölken, Martin Stevens

**Affiliations:** 1 Department of Health Services Research, School of Medicine and Health Sciences, Carl von Ossietzky Universität Oldenburg, Oldenburg, Germany; 2 Department of Orthopedics, University of Groningen, University Medical Center Groningen, Groningen, The Netherlands; 3 Division of Orthopedics at Campus Pius-Hospital, School of Medicine and Health Sciences, Carl von Ossietzky Universität Oldenburg, Oldenburg, Germany; 4 Department of Health Sciences, University of Groningen, University Medical Center Groningen, Groningen, The Netherlands; 5 Reha-Zentrum am Meer, Bad Zwischenahn, Germany

**Keywords:** hip replacement; knee replacement; revision arthroplasty; care pathway; qualitative research

## Abstract

This study aimed to learn from the experiences of patients who have undergone medical rehabilitation following hip or knee arthroplasty in the Netherlands and Germany. A qualitative focus group study was conducted among Dutch revision total hip and knee arthroplasty patients who had experience with initial rehabilitation in the Netherlands plus inpatient rehabilitation in Germany as part of the ‘Common Care’ pilot project. The focus group was recorded, transcribed verbatim and pseudonymised. A thematic analysis using an inductive approach was performed. Five patients and two family members joined the focus group. Analysis revealed five themes: language, location, therapy content, outcomes and structure. Content-wise, therapy, outcomes and structure of rehabilitation in Germany were perceived as more beneficial. In terms of location and language, patients favoured the Dutch approach. Participants generally expressed higher levels of satisfaction with the German rehabilitation trajectory versus the Dutch. The German approach’s main advantages were the more extensive therapy content and supervision plus a feeling of faster recovery, the Dutch were the home-sweet-home feeling, language and travel distance. Future research should further evaluate effects of rehabilitation policies especially on return to work.

**Supplementary Information:**

The online version contains supplementary material available at 10.1186/s13104-026-07763-0.

## Introduction

Rehabilitation services for patients following hip or knee arthroplasty differ between Germany and the Netherlands. Dutch patients are generally sent home with a recommendation for physiotherapy following a 2–3 day hospital stay [1]. As physiotherapy is not reimbursed by Dutch basic health insurance [2], most patients have additional insurance for a specified number of sessions [3]. By contrast, after a week-long hospital stay, German patients can attend a 3-week rehabilitation programme at a specialised rehabilitation centre, and additional physiotherapy can be prescribed when considered indicated [4].

In recent decades more countries have implemented Enhanced Recovery After Surgery policies including a home-discharge policy [5]. Research on outcomes suggests non-inferiority of self-responsible home rehabilitation versus clinic-based or inpatient rehabilitation [6–8]. Similarly, comparison between the German and Dutch approaches after primary total hip arthroplasty (THA) showed no statistically significant difference in patient-reported outcomes or satisfaction 6 and 12 months postoperatively [9]. Another German-Dutch comparison in a working population showed that German primary THA patients had better functional outcomes and satisfaction than their Dutch peers at three and six months postoperatively, with faster return to work (RTW) [10].

The Interreg-V-funded project ‘Common Care’ (#201186) was initiated in 2018 in the Ems-Dollard region aiming to develop sustainable cross-border healthcare infrastructure [11]. Part of ‘Common Care’ was a pilot project in which Dutch working-age patients undergoing (revision) THA or total knee arthroplasty (TKA) in the Netherlands got the opportunity to rehabilitate at a German rehabilitation centre. As the patients had prior experience with postoperative rehabilitation in the Netherlands, they were deemed able to make a fair comparison between the German and Dutch approaches. Aim of this study is to learn about their experiences with both rehabilitation approaches, to better understand the strengths and weaknesses of the German and Dutch systems.

## Methods

### Design

A qualitative focus group study among Dutch (revision) THA and TKA patients who received inpatient rehabilitation in Germany within the above-mentioned pilot project. The focus group method was selected for its ability to facilitate participants’ expression of ideas and experiences, contributing to a more comprehensive understanding of end-users’ attitudes, experiences, and expectations [12].

### Recruitment

Five participants joined the pilot. All had primary surgery and rehabilitation in the Netherlands and secondary surgery at the Orthopaedics Department of University Medical Center Groningen (UMCG), the Netherlands, then attended the rehabilitation programme at Reha-Zentrum Am Meer in Bad Zwischenahn, Germany, between December 2021 and June 2022. In December 2022, the participants and their partners were invited to join the focus group.

### Focus group

The focus group met at UMCG on 18 January 2023. It lasted two hours and was moderated by two researchers experienced in conducting focus groups but not involved in ‘Common Care’ (MA, YM). Assisting and providing additional information were one researcher (MS), a specialised nurse (AK) and a physician assistant (MJK) who had been involved in ‘Common Care’. Participants received travel cost reimbursement and a gift card.

### Interview guide

A semi-structured interview guide was used (Supplementary file 1). All participants were asked to first write down advantages and disadvantages they experienced using the Dutch and German rehabilitation services on sticky notes. The notes were then arranged on a whiteboard, serving as starting point for the discussion. More in-depth explanations and experiences regarding these advantages and disadvantages were shared during the interview, and additional ones were mentioned. Last, it was asked what each country should take away from the other’s approach.

### Data analysis

The focus group was recorded, transcribed verbatim, and pseudonymised following established standards [13]. A thematic analysis was performed using an inductive approach by YM, who generated initial codes and developed themes after becoming acquainted with the data. MS and GHS reviewed these themes and discussed them to establish the final code definitions as described by Christou [14]. Besides establishing themes, five categories were created: ‘Advantages Netherlands’, ‘Disadvantages Netherlands’, Advantages Germany’, ‘Disadvantages Germany’, and ‘Other’. Themes could appear in multiple categories.

### Ethics

All patients provided written informed consent. The study was approved by the UMCG Institutional Review Board (MET2021/601).

## Results

**Fig. 1:**
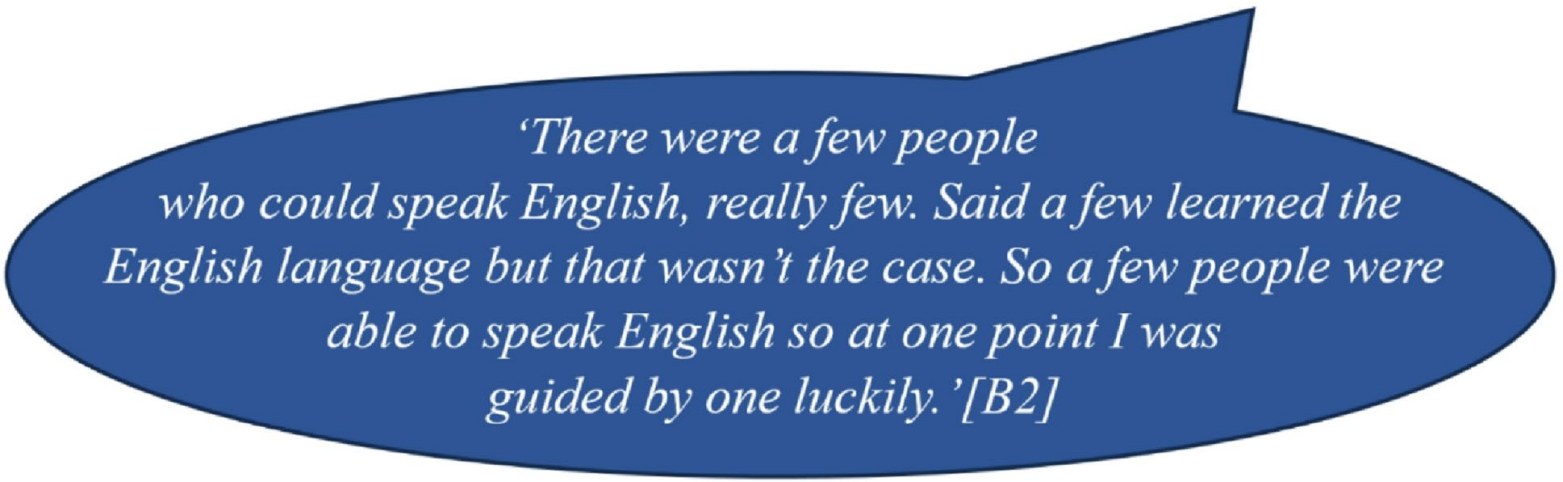
Example quotation language theme

**Fig. 2:**
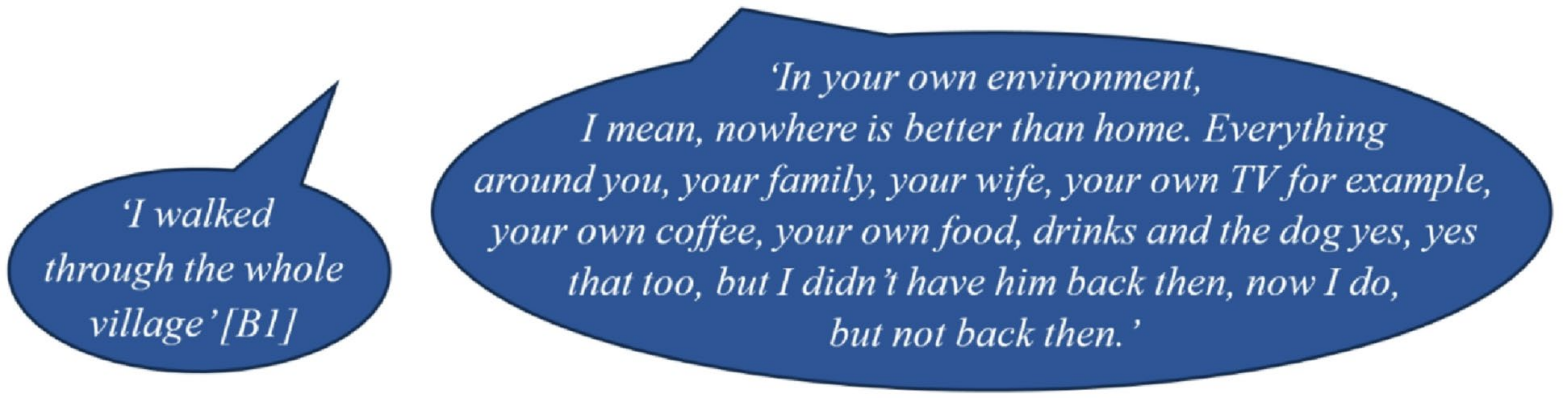
Example quotations location theme

**Fig. 3:**
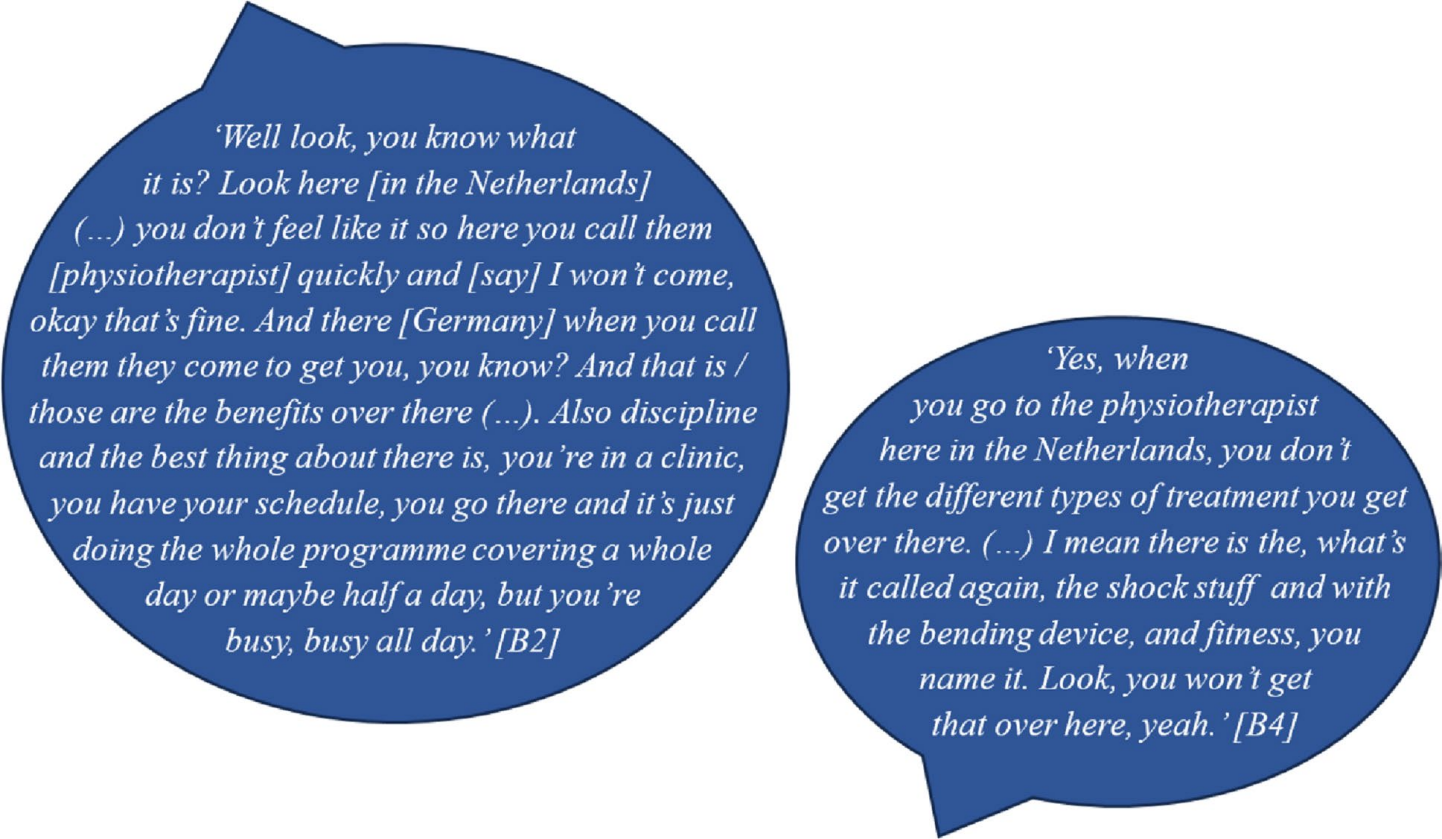
Example quotations therapy intensity and content theme

**Fig. 4:**
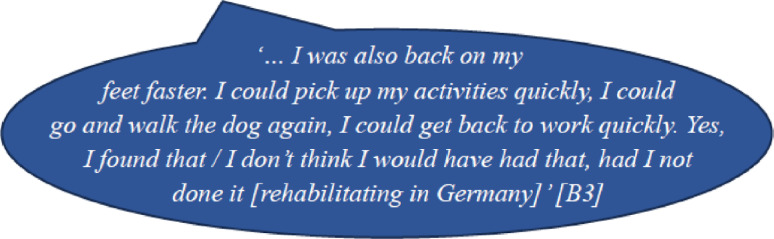
Example quotation outcome theme

**Fig. 5:**
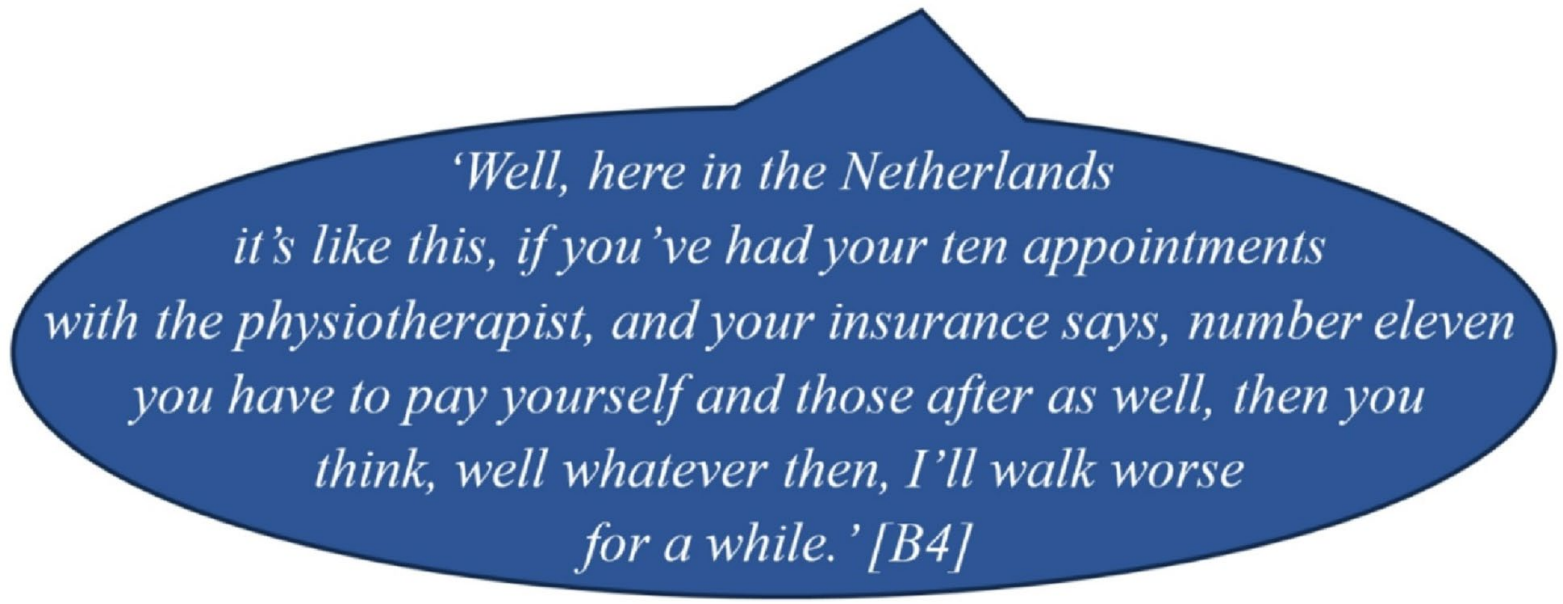
Example quotation organizational structure theme

All five patients from the ‘Common Care’ pilot project were willing to join the focus group, and two brought a family member, resulting in seven participants. While the primary discussion was between patients, family members added specific aspects if presumed relevant. Table 1 shows participants’ demographic information. Analysis revealed five themes: language, location, therapy content, outcomes, and structure. Figures 1, 2, 3, 4 and 5 show example quotations. Supplementary file 2 presents an overview of the themes, subthemes, categories, and supporting quotations.

**Table 1:** Focus group participants’ demographic and surgical characteristics

	Role	Age	Sex	Time since first surgical procedure (months)	Time since last surgical procedure (months)
B1	Patient	57	F	375	8
B2	Patient	67	M	97	13
B2P	Family member (wife)	unk.	F	NA	NA
B3	Patient	54	M	~ 91	8
B4	Patient	54	M	163	8
B5	Patient	71	F	62	9
B5P	Family member (son-in-law)	unk.	M	NA	NA

*Unk* unknown, *F* female, *M* male, *NA* not applicable

### Theme 1: Language

The most frequently stated advantage of rehabilitating in the Netherlands was communication in the native language. Language was mentioned as a disadvantage of rehabilitating in Germany, where English was not as widely spoken to communicate effectively. The ability to speak German proved valuable for those able to, others found a buddy able to speak English or Dutch to help out.

### Theme 2: Location

Both locations had advantages and disadvantages, with the predominant Dutch advantage and German disadvantage being the home-sweet-home feeling and being able to continue one’s normal activities. Still, the new environment provided a possibility to explore and enjoy the surrounding area, stimulating activity.

Another advantage of staying at home was being cared for by family instead of strangers, although this also led to a higher burden for caregiving family members. Another subtheme, especially relevant for family members, was travel distance and visits to the rehabilitation centre. Participants were generally happy with the support of the individual healthcare providers in Germany, who motivated and gave them confidence, yet would have valued slightly more support the first few days to facilitate better orientation. Two participants who coincided at the facility benefited from each other’s support.

Other topics addressed included improved ability to work from home compared to working from the facility; food quality and atmosphere, with both positive and negative ratings; and the method of transport to the facility as a point for improvement.

### Theme 3: Therapy intensity and content

Patients noticed several differences regarding content and executions of therapeutic interventions received. Discipline and intensity of the German approach were primarily mentioned as advantageous over the Dutch, although some participants perceived it as perhaps slightly too intense.

Moreover, in Germany exercises varied more and a greater range of activities was allowed. Measures included activities with a continuous passive motion machine, skateboards, use of a swimming pool, and manual lymphatic drainage. The more central organisation of rehabilitation led patients to perceive healthcare providers in Germany as more knowledgeable than their Dutch counterparts. One measure, provided only in the Netherlands, was psychological support aimed at enhancing pain-coping strategies. Only one participant had received psychological support, and deemed it very beneficial, leading to the question of why something similar was not offered in Germany, especially as the facility employed psychotherapists.

Several participants mentioned another advantage of the German approach: always having a healthcare professional nearby, whereas in the Netherlands patients have to travel to a healthcare facility. Besides, wound care is given greater attention in Germany. Last, participants mentioned minor practical differences, such as types of medication and needles used.

### Theme 4: Outcomes

Generally, patients were highly satisfied with the outcomes in Germany. The vast majority had the feeling they achieved better short-term results compared to their prior rehabilitation in the Netherlands, especially in terms of walking.

### Theme 5: Organizational structure

The major organizational difference was treatment financing. While in the Netherlands basic health insurance does not include physiotherapy, Dutch participants learned their German counterparts could easily receive additional treatments if necessary, supplementary to their time at the rehabilitation centre.

Other subthemes were the planning and information about the rehabilitation trajectory, and communication from and between stakeholders.

## Discussion

The current study aimed to gain insight into experiences of patients who underwent rehabilitation following (revision) THA/TKA both in the Netherlands and Germany, to gain a more nuanced understanding of strengths and weaknesses of both policies. Participants expressed high levels of satisfaction with the treatment provided in Germany, particularly compared to their previous experience with the Dutch approach, and appreciated the more extensive content and supervision. Most perceived the more intense German approach to benefit their recovery and facilitate return to daily activities. Participants also identified advantages of the Dutch approach, which were primarily related to the home-sweet-home feeling, language, and travel distance.

Therapy content between both countries differed considerably in multiple aspects. Participants tended to prefer the more extensive German approach: its higher intensity, supervision, and discipline made them perceive they were in good hands and recovered faster. This, however, is not shown in some literature findings that suggest non-inferiority of home versus inpatient rehabilitation regarding clinical outcomes [6–8]. However, those outcomes were measured only starting at ten weeks post-surgery, while best improvements can be expected on a shorter term directly after finishing inpatient rehabilitation. Faster recovery in the first weeks is especially important for working-age patients. A Dutch-German comparison showed that, on average, German patients resume work 2.3 weeks earlier than Dutch patients [10]. With the rising provision of THA/TKA at a younger age RTW time becomes an increasingly relevant outcome measure.

Location-wise, participants generally favoured home rehabilitation, as this gives a better opportunity to continue or pick up one’s preferred activities, including work. Besides, shorter travel distance was a relevant factor, particularly for family members. On the other hand, home rehabilitation increases the burden on family members and might reduce focus on rehabilitation. The latter was not perceived as problematic because of the low intensity of rehabilitation offered in the Netherlands. Still, at the German rehabilitation centre participants had some spare time, and the pleasant environment stimulated going for a walk, which might positively influence recovery compared to relaxing or working at home.

Structure and reimbursement differ considerably between these countries’ rehabilitation approaches. Where in the Netherlands no reimbursement is provided by basic health insurance, in Germany costs for the 3-week inpatient rehabilitation are reimbursed, as is additional physiotherapy if needed. For patients still in the workforce, the German pension insurance (Deutsche Rentenversicherung) additionally offers two intensified aftercare services – IRENA and T-RENA – following discharge from the facility [4]. Without usage of these programmes, one participant already perceived faster RTW following rehabilitation in Germany. While the emphasis during the focus group was on therapy content, and less regarding the costs or reimbursement, one participant reported preferring a longer recovery time over paying for additional physiotherapy. However, this may not even be an option due to their financial situation, resulting in no supervision at all during their rehabilitation. Another structural difference is the centralisation at German rehabilitation facilities versus differentiated individual physiotherapy practices in the Netherlands. This might explain why participants perceived the German healthcare professionals as more knowledgeable.

### Strengths and limitations

This study included a unique patient population that experienced both the Dutch and German approaches to rehabilitation following (revision) THA and TKA. Due to the pilot project’s small sample, only five patients were eligible for this study. Consequently, it is possible that data saturation was not reached. Additional participants may have added other experiences. The number of five patients, however, was due to financial constraints within the ‘Common Care’ project.

Participants stayed at the German rehabilitation center during the Covid-19 breakout. While this presumably did not directly influence received treatments, it did affect the social context. Arrival at the facility was not considered very welcoming—probably because of the strict pandemic regulations. Besides, family members were not welcome, and social interaction with fellow patients was limited, possibly leading to a lower sense of support.

The pilot characteristic of this exchange study should also be considered in terms of organizational aspects. Several teething problems were experienced for which participants gave recommendations like improving communication between the different participating healthcare institutions and between healthcare institutions and patients. Patients likewise suggested ensuring that German and Dutch patients arrive at the rehabilitation centre at the same postsurgical timepoint, as they generally went there a few days earlier than their German peers, resulting in a different entry level.

## Conclusion and future implications

Participants expressed higher levels of satisfaction with the German rehabilitation trajectory compared to the Dutch. The German approach’s main advantages were content, intensity, and supervision of therapy, leading to a feeling of faster recovery. The main Dutch approach’s advantages were the home-sweet-home feeling, their own language, and shorter travel distance. However, for a more profound and comprehensive understanding, additional focus groups are required, as the current data most likely has not reached data saturation. In addition, future research should focus on rehabilitation policies’ effects on RTW time for the growing group of working-age patients. It can be hypothesised that a more extensive rehabilitation, as offered in Germany, could especially be beneficial for Dutch patients returning to work after (revision) THA/TKA. Furthermore, more research into the influence of health system factors on outcomes and satisfaction following THA and TKA would increase the understanding on this topic and help to optimize the rehabilitation process.

## Supplementary Information

Below is the link to the electronic supplementary material.

Supplementary Material 1.

Supplementary Material 2.

## Data Availability

The datasets are available from the corresponding author on reasonable request.

## Abbreviations

THA: Total hip arthroplasty
TKA: Total knee replacement
RTW: Return to work

## References

[1] Oosting E, Kapitein PJC, de Vries SV, Breedveld E. Predicting short stay total hip arthroplasty by use of the timed up and go-test. BMC Musculoskelet Disord. 2021;16(1):361. 10.1186/s12891-021-04240-6. 10.1186/s12891-021-04240-6 PMC8052837 33863323

[2] RIVM. Zijn fysiotherapie en oefentherapie opgenomen in het basispakket? 2024. https://www.rijksoverheid.nl/onderwerpen/zorgverzekering/vraag-en-antwoord/is-fysiotherapie-opgenomen-in-het-basispakket. Accessed 10 Apr 2024

[3] Hofstede SN, Vliet Vlieland TP, van den Ende CH, Nelissen RG, Marang-van de Mheen PJ, van Bodegom-Vos L. Variation in use of non-surgical treatments among osteoarthritis patients in orthopaedic practice in the Netherlands. BMJ Open. 2015;9(9):e009117. 10.1136/bmjopen-2015-009117. 10.1136/bmjopen-2015-009117 PMC4567674 26353874

[4] Deutsche Rentenversicherung. Reha-Nachsorge. Deutsche Rentenversicherung 2024. https://www.deutsche-rentenversicherung.de/DRV/DE/Reha/Reha-Nachsorge/reha-nachsorge_node.html. Accessed 15 Apr 2024.

[5] Ljungqvist O, Scott M, Fearon KC. Enhanced recovery after surgery: a review. JAMA Surg. 2017;152(3):292–8. 10.1001/jamasurg.2016.4952. 28097305 10.1001/jamasurg.2016.4952

[6] Buhagiar MA, Naylor JM, Harris IA, Xuan W, Adie S, Lewin A. Assessment of outcomes of inpatient or clinic-based vs home-based rehabilitation after total knee arthroplasty: a systematic review and meta-analysis. JAMA Netw Open. 2019;2(4):e192810–192810. 10.1001/jamanetworkopen.2019.2810. 31026026 10.1001/jamanetworkopen.2019.2810 PMC6487570

[7] Naylor JM, Hart A, Mittal R, Harris I, Xuan W. The value of inpatient rehabilitation after uncomplicated knee arthroplasty: a propensity score analysis. Med J Aust. 2017;207(6):250–5. 10.5694/mja16.01362. 28899328 10.5694/mja16.01362

[8] Onggo JR, Onggo JD, De Steiger R, Hau R. The efficacy and safety of inpatient rehabilitation compared with home discharge after hip or knee arthroplasty: a meta-analysis and systematic review. J Arthroplasty. 2019;34(8):1823–30. 10.1016/j.arth.2019.04.001. 31053467 10.1016/j.arth.2019.04.001

[9] Füssenich W, Gerhardt DM, Pauly T, et al. A comparative health care inventory for primary hip arthroplasty between Germany versus the Netherlands. Is there a downside effect to fast-track surgery with regard to patient satisfaction and functional outcome? HIP Int. 2020;30(4):423–30. 10.1177/1120700019876881. 31505973 10.1177/1120700019876881

[10] Wijnen A, Seeber GH, Dietz G, et al. Effectiveness of rehabilitation for working-age patients after a total hip arthroplasty: a comparison of usual care between the Netherlands and Germany. BMC Musculoskelet Disord. 2023;2023/06/27(1):525. 10.1186/s12891-023-06654-w. 10.1186/s12891-023-06654-w PMC10294515 37370054

[11] INTERREG, Common Care—HOCHWERTIGE, UND WOHNORTNAHE GESUNDHEITSVERSORGUNG GRENZÜBERSCHREITEND IN DER EDR. Gemeinsames INTERREG-Sekretariat bei der Euregio Rhein-Waal. https://interregv.deutschland-nederland.eu/en/project/common-care/. Accessed 11 Apr 2024.

[12] Kitzinger J. Focus groups. Qualitative research in health care; 2006. p. 21–31.

[13] Karbach U, Fuß S. Grundlagen der Transkription; 2014.

[14] Christou PA. How to use thematic analysis in qualitative research. J Qual Res Tourism. 2023;1(2):1–17. . 10.4337/jqrt.2023.0006

