# Supplementary material for: German rehabilitation after total hip or knee arthroplasty through Dutch eyes: a qualitative focus group pilot study
Source: BMC Res Notes. 2026 Mar 13;19:163. doi: 10.1186/s13104-026-07763-0 (PMC13064102; doi:10.1186/s13104-026-07763-0)
Supplement: Supplementary file 1 — Supplementary Material 1. [file 13104_2026_7763_MOESM1_ESM.docx]

**Hip Across**

**Interview guide for a focus group with Dutch patients who underwent rehabilitation in Germany following revision of a total hip replacement**

**General information**

| Objective | The aim of this focus group is to find out more about how Dutch patients experienced rehabilitation care at the German rehabilitation center in Bad Zwischenahn. The target group consists of Dutch patients who underwent primary knee or hip replacement in the Netherlands and rehabilitated in the Netherlands, but who went to Bad Zwischenahn in Germany for their rehabilitation after revision hip or knee replacement. The questions we hope to answer with the focus group are primarily what people experienced as the biggest differences between the healthcare systems, and how these differences influenced the rehabilitation process. Secondarily, we want to find out what motivated them to accept the offer to undergo rehabilitation in Germany, and learn more about the strengths and weaknesses of both methods of rehabilitation. In addition, we hope that participants will be able to identify areas for improvement in both healthcare systems. |
| --- | --- |
| Duration of the interview | 90-120 min |
| Location | University Medical Center Groningen (UMCG) |
| Preparation | Coffee and tea; Post-it notes; Flipchart + markers |

**Part 1: Opening, Introduction to the project, Preparation**

| Opening | Welcome, introduction of the interview team. |
| --- | --- |
| Introduction to the project | Before we start with the focus group, we would like to briefly explain the purpose of the focus group.  We are interested in how the differences in the organization of rehabilitation after hip or knee replacement in the Netherlands and Germany work out in practice. Because you have undergone rehabilitation in both the Netherlands and Germany, we are curious about your experiences with both methods of rehabilitation. We are also interested in your reasons for accepting the offer to undergo rehabilitation in Germany.  The interview will take approximately 2 hours. There will be a short break about halfway through. |
| Recording and data security | The interview will be recorded with an audio recording device. Later, the interview will be transcribed, pseudonymized, and analyzed. Everything you say will be treated confidentially, which means that no one outside this project will know that it was you, or which statements came from you.  Participation in this interview is voluntary, and you may indicate at any time that you wish to stop. You are also not required to answer any questions if you do not wish to answer. There are no right or wrong answers to any of the questions. If you have any questions during the interview, you are welcome to ask them at any time. I would also ask you not to mention other people's names in any of your answers; use terms such as "patient" or "my brother" instead.  Important, everything discussed here is confidential. Please do not share what has been discussed with others around you. Treat each other with respect, let each other finish speaking, and allow space for others' opinions.  Do you have any further questions about the information I/we have just provided you with?  Finally, I would like to ask you to turn off your cell phone to prevent problems with the recording equipment. |
| End of the opening  Recording | Do you have any questions?  Then I/we will now start the recording. |

**Part 2: Start of the interview**

| **Topic** | **Key questions** | **In-depth questions** |
| --- | --- | --- |
| **Welcome** | Sticky-notes and a flipchart are available. During the coffee/tea time, on the sticky-notes, participants can write down the various expectations they had beforehand regarding the treatment and outcomes of (1) the first joint replacement and rehabilitation in the Netherlands and (2) the second joint replacement and rehabilitation in Germany. | |
| **Introduction** | - We would like to ask you to briefly introduce yourselves. | - What was the situation like before the joint replacement? - How long ago was the first joint replacement surgery? - What was the reason for the revision? - How long ago was the revision? - What were your main goals prior to your primary joint replacement? |
| **Rehabilitation in the Netherlands** | - You underwent rehabilitation in the Netherlands after your primary hip or knee replacement. How did you experience this type of rehabilitation at the time? | - What were your expectations regarding rehabilitation? - How did these expectations turn out? - To what extent did you receive help from healthcare professionals during rehabilitation, and to what extent did you rehabilitate independently/at home? Why? - How do you look back on your rehabilitation in the Netherlands (without comparing it to rehabilitation in Germany)? |
| **Motivation** | - At some point, you were asked whether you wanted to undergo rehabilitation in Germany as part of the “Common Care” project. What considerations played a role in your decision? |  |
| **Rehabilitation in Germany** | - How did you experience rehabilitation in Germany? | - What were your expectations regarding rehabilitation in Germany (possibly in comparison with the process in the Netherlands/sticky notes on the board)? - How did these expectations turn out? - How did this rehabilitation look like for you? - How do you look back on the rehabilitation in Germany? |
| **Break** | Sticky-notes and a flipchart are available. During the coffee/tea break, participants can place the sticky notes on the flipboard listing the strengths and weaknesses of both rehabilitation methods. | |
| **Differences between rehabilitation in the Netherlands and Germany** | - What differences did you experience between rehabilitation in the Netherlands and in Germany? - What did you consider to be the strengths and weaknesses of both rehabilitation methods? | - How do you think these differences/strengths/weaknesses influenced the differences in outcomes in terms of joint function? - How did these differences affect your daily activities? - How did these differences affect your satisfaction? - What could the Netherlands learn from the German approach to improve the process? - What could Germany learn from the Dutch approach to improve the process? |
| **Optimal rehabilitation** | - With your experience of both methods of rehabilitation, what do you think optimal rehabilitation after a hip or knee replacement would look like? |  |
| **Wrap up** | We have now reached the end of the interview.  We would like to thank you once again for your participation!  If you have any questions now or later, please feel free to ask them. | |
