# Supplementary material for: German rehabilitation after total hip or knee arthroplasty through Dutch eyes: a qualitative focus group pilot study
Source: BMC Res Notes. 2026 Mar 13;19:163. doi: 10.1186/s13104-026-07763-0 (PMC13064102; doi:10.1186/s13104-026-07763-0)
Supplement: Supplementary file 2 — Supplementary Material 2. [file 13104_2026_7763_MOESM2_ESM.docx]

| Theme | | Quote | Category |
| --- | --- | --- | --- |
| Theme 1: Language | | |  |
|  | Subtheme: Dutch is easy as it is the native language | |  |
|  |  | ‘Well, Dutch, it’s easy.’ [B1] | AN |
|  | Subtheme: Limited English knowledge on German side | |  |
|  |  | ‘Well, that not really came true.’ [B4] | DG |
|  |  | ‘They were very happy if you were able to speak German’ [B1] | DG |
|  | Subtheme: Solutions for language barrier | |  |
|  |  | ‘I did not feel like a stranger there. Yes, talked German quite easily, I made contact quite easily. So I / yes, I actually felt quite comfortable there.’ [B3] | O |
|  |  | ‘There were a few people who could speak English, really few. Saying eh, there are a few nurses that learned the English language but that was not the case. So a few people were able to speak English so at one point I was guided by one luckily.’[B2] | O |
|  |  | ‘But when I came in there [rehabilitation center], when I registered, I did indicate that I don’t speak German, and they instantly adjusted it for me, so in that regard, if they realize at the intake whether you speak German or not, they can arrange it there.’ [B2] | O |
|  |  | ‘There were a few who Dutch / My physiotherapist also spoke Dutch.’ [B3] | O |
| Theme 2: Location | | |  |
|  | Subtheme: Home-sweet-home | |  |
|  |  | ‘In your own environment, I mean, nowhere is better than home. Everything around you, your family, your wife, your own TV for example, your own coffee, your own food, drinks and the dog yes, yes that one too, but I didn’t have him back then, now I do, but not back then.’ [B3] | AN |
|  |  | ‘I can remember December 24^th^, they had a meeting in the canteen. All the patients came to the (...?). And at one point, yeah I was struggling a bit, so I didn't go. And um, I was back to my bed and just crying like a little boy there. Because I missed my family and everything, Christmas time. And at one point I got a phone call, where are you? And I then explained that and within five minutes four people were standing in front of me, nurses to talk to me and to give me a peptalk. [B2] | DG |
|  | Subtheme: Enjoyable environment | |  |
|  |  | ‘Well, if you have a nice view over the lake where the boats are sailing (…). That was quite nice.’ [B4] | AG |
|  |  | ‘I walked through the whole village’ [B1] | AG |
|  | Subtheme: Burden on family | |  |
|  |  | ‘That you, yes I mean that you than always have to ask your partner, that is what I mean. To help you. And the advantage is that it is your own partner, but if you are in the Netherlands, and you are home, and you need help. Then you always have to ask your partner, whilst they also have their own duties, or also has to work. Or you have to ask your children. I had to ask my 9 year old daughter whether she could make a sandwich for me. Yes, I sometimes find that objectionable. Every cup of coffee you ask for.’ [B3] | AN+DN |
|  |  | ‘Yeah, I was an informal caregiver, and besides I had to work as well. So yeah, that was quite rough.’ [B2P] | DN |
|  | Subtheme: Travel distance | |  |
|  |  | ‘Well, the advantage is of course, you can skip the driving part, so you do not have to combine anything, consult in terms of work, and so on and so forth. (…) That’s actually, for me I think the only advantage I can think of. Driving distance and scheduling in terms of work. Germany was an hour and a half drive, two hours, I don’t even remember.’ [B5P] | AN |
|  |  | ‘It was a process indeed no, how you drop him, and you hear nothing for a while,, I got no communication nothing. And at one point he needs for example needed swimming shorts, and I was like, how do I do that, should I drive there, but then again that was not allowed, so I um, yes we went after the postal address and such. Then I posted it.’ [B2P] | O |
|  |  | 'And then you have your knee, you have your leg straight, and then you have to get in the car, then you have to go to Germany. Well has not been a nice ride. I can tell you that' [B5P] | DG |
|  |  | ‘I had prepared myself for that, well my wife is here (Netherlands) as well, so I could get there less easily, but yeah, then that Covid came on top of it and then it became even more difficult’ [B3] | DG |
|  | Subtheme: Atmosphere | |  |
|  |  | ‘It really is a factory. So If you can get in there as a new patient in the covid time, it is already an accomplishment if you manage to, ehm yes, it all goes through on the conveyor belt. There is / It is unfriendly, it goes very fast and you have to understand. I thing that is because of the size of the place. If it were half the size it might all be more relaxed, more convivial. But now it all has to go on and on.’ [B5P] | DG |
|  |  | ‘Except for the treatment during the welcome. After that it was, I found it totally normal, they were nice and friendly. There were a lot of nice people around.’ [B3] |  |
|  | Subtheme: Work | |  |
|  |  | ‘Yes, when you are home you are working. Yes, then you can work from home. That is an advantage, yes. You can at least raise a few pennies.’ [B4] | AN |
|  |  | ‘The Wi-Fi over there (rehabilitation center) was not great. But when you had Wi-Fi, then yeah, you could do something. But less then when I had rehabilitated at home. Yeah that for sure.’ [B4] | DG |
|  | Subtheme: Support | |  |
|  |  | ‘The physiotherapist was very helpful to me. (…) She was really like everything will be fine, and maybe it is going a bit down now, but pay attention and eh ‘Wir schaffen das’. But yes, she pulled me though.’ [B3] | O |
|  |  | ‘Yeah, but I also think it was a disadvantage because of covid, because if you would have been able to sit together at a table and discuss and then, then yeah, it was a totally different time I think.’ | O |
|  |  | ‘And you also always can directly contact if you just, this hurts so much, or I cannot manage this, and ah, then they encourage you or (…) try this or that, try to think about this. You do not have that at home of course. There you are alone with your wife and family.’ | AG + DN |
|  |  | ‘Kind of shared sorrow is half sorrow.’ [about rehabilitating together with a fellow Dutch participant] [B3] | O |
|  | Subtheme: Food | |  |
|  |  | ‘I sent pictures to my husband every day. This is it for today, and this for this day. (…) But I understand, I do understand it, because there are of course also people with a diet or whatever. That everything had to lean. But I you just get broth, with a few potatoes and vegetables, and that is the meal. Then I think, okay?’ [B5] | DG |
|  |  | ‘I just talked about it (food) with her, because I liked the food. And she had food poisoning, she told me’ [B2] | O + DG |
|  |  | ‘I ate many salads over there [rehabilitation center], because the other food I was not so sure about.’ [B3] | DG |
| Theme 3: Therapy content | | |  |
|  | Subtheme: Intensity and discipline | |  |
|  |  | ‘Well look, you know what it is? Look here, and you don’t feel like it, you know how it goes, it’s in your head, you don’t feel like it so here you call them shortly and, I won’t come, okay that is fine. And there when you call then they come and get you, you know? And that is eh / Those are the benefits over there (…). Also discipline and the best thing about there is, you are in a clinic, you have your schedule, you go there and it is just doing the whole program covering a whole day or maybe half a day, but you are busy, busy all day.’ [B2] | DN + AG |
|  |  | ‘I did find the transition for the Netherlands to Germany a bit / like here you are lying down than you have surgery, the physio comes once to have a look here in the hospital. Well, just a bit of exercise or whatever, and you arrive over there and you have to get going immediately, pain or no pain, let’s go, you go for it.’ [B5] | O + DG |
|  |  | ‘You go and work you know, look here in the Netherlands you got to therapy for an hour once is a while, and then you go back home and, then you are really busy with yourself.’ [B2] | DN + AG |
|  |  | ‘But also intensive, right, the whole day long. And you have that with physiotherapy, you have that here in the Netherlands, of course not if you go to a rehabilitation center, but twice a week, if you are lucky, some only once a week. Well, that, sorry to say it, but you can’t make that. In terms of muscle strength and so on. Then you would need to have a lot of self-discipline, I think. To do exercises at home as well. And who has that?’ [B1] | AG + DN |
|  |  | ‘Well, I have been to the physiotherapist here [Netherlands] as well, but yeah, that is not fun as well. Well I mean that is not pain free as well. And otherwise, yeah in Germany it is just different, I am busy all day all hours. That is just very different.’ [B4] | O |
|  |  | ‘When I received therapy here eight years ago, the therapist came to my home and asked me (…) what do you want to achieve this week? I think, I want to go up those stairs, I want to shower you know, because I spent a few months just using a flannel, fount it a bit difficult, but the staircase has thirteen steps, so I spent two weeks, every day a step up and a step down. And there in Germany it is just the whole staircase up at once.’ [B2] | O |
|  |  | ‘And then you have some time in between, and then they say, yeah but you have your skateboard in our room, go and sit on it for twenty minutes.’ [B1] | O |
|  |  | ‘Well, actually starting, from Germany I am actually, yeah, maybe it is my mage, I don’t know, I collapsed a bit. Physically, yes. Just, my whole body was exhausted.’ [B5] | DG |
|  |  | ‘Yeah, I think I did find the first week there the / actually kind of the hardest. You arrived there, you basically had to do everything yourself, shower, I don't know what. Well, I found that quite intense.’ [B4] | O |
|  | Subtheme: Therapy content and knowledge | |  |
|  |  | ‘Yes, when you go to the physiotherapist here in the Netherlands, you do not get the different types of treatment you get over there. (…) I mean there is the, eh what is it called again, the shock stuff and with the bending device, and fitness, and you name it. Look, you won’t get that over here, yes.’ [B4] | DN + AG |
|  |  | ‘Yes, I feel like they just have a lot of knowledge. (…) And that gave me a very good feeling, like, I am in good hands. What can happen to me now?’ [B3] | AG |
|  |  | ‘So I knew from Germany that I had to go to the physiotherapist here in (the local town) the very next week. So I had arranged that in advance. But eh, they said / they were really shocked about what I had to do (in Germany).’ [B5] | O |
|  |  | ‘What I noticed was that the German doctor, they were more afraid of using paracetamol for an extended timeframe than oxycodone. (…) Here in the Netherlands it’s kind of the other way around.’ [B3] | O |
|  |  | ‘The needles. Those are way too thick. And they immediately said, yes we don’t have the needles you have in the Netherlands. Yes, that is correct.’ [B5] | AN |
|  |  | ‘And they were looking at your wound. That was the first thing you had to do, to the wound nurse.’ [B1] | AG |
|  |  | ‘But also that you, I mean, you saw a lot of people with new knees, and new hips. That you ended up in the right place, I always had that feeling of I'm good here. I have confidence in this. They know what they are doing. That did give me a lot of, yes, may sound a bit woolly, but a lot of strength. To persevere.’ [B3] | AG |
|  |  | ‘But well, you were also busy with physiotherapy and that kind of stuff. Swimming and I don’t know what.’ [B4] | AG |
|  |  | ‘I believe the therapy you receive over there in Germany is really personalized no? It is not general therapy. You have something with you knee so we do this. You have something with your hip so that will be adapted.’ [B2] | AG |
|  |  | ‘My knee was very thick and then they do lymphatic drainage. Here in the Netherlands I believe that they find that nonsense.’ [B3] | AG |
|  |  | ‘Yeah, they also have special trails over there [rehabilitation center].’ |  |
|  |  | ‘If you look in Germany, your leg is then placed unloaded in such a leg machine. I didn't experience that here in the Netherlands. The first time I had surgery I could bend my leg much later than now.’ [B3] | AG |
|  |  | ‘Yeah maybe was, we maybe, we did not receive it in Germany, but some psychological support. How do you deal with, for example, pain. I think that is something to think about. No did not receive it in Germany. I have done something like pain rehabilitation first. And then you learn to deal with pain. That it does not have to be a limitation.’ [B3] | AN |
|  | Subtheme: Supervision | |  |
|  |  | ‘That you get intensive supervision there. They carry you through. Here you have a physiotherapist once or twice a week. If something happens, you have to call the doctor, or have to go to the emergency department, or to the medical center. I experienced that with my previous surgery, it is a drama. Here [in Germany] you always have people close.’ [B3] | DN + AG |
|  |  | ‘I would like to bring over the whole concept to us (in the Netherlands). Really everything. That there is a doctor around, a wound care nurse, the physiotherapy. Really everything’. [B1] | AG |
|  |  | ‘And you also always can directly contact if you just, this hurts so much, or I cannot manage this, and ah, then they encourage you or (…) try this or that, try to think about this. You do not have that at home of course. There you are alone with your wife and family.’ | AG + DN |
| Theme 4: Outcomes | | |  |
|  | Subtheme: Time to recovery | |  |
|  |  | ‘That you can remove the crutches sooner. (…) That you sooner just, yes just walk. Get confidence. I mean within a week I had to go up the stairs over there, well you don’t have to try that here.’ [B4] | AG |
|  |  | ‘So the first two times I was at home, I walked without crutches. Now almost a year has passed and I still walk with one crutch. Despite the fact that I went to Germany.’ [B5] | DG |
|  |  | ‘And I was also back on my feet faster. I could pick up my activities quickly, I could go and walk with the dog again, I could get back to work quickly. Yes, I found that / I don’t think I would have had that if I had not done it [the rehabilitation in Germany]’ [B3] | AG |
|  |  | ‘Last year I could bend my knees about 25 to 30 degrees, and there they have this continuous passive motion machine, so there I could (…) So yeah I went to Germany and then I could 75 degrees and now I can do about 100 degrees, so it improved a lot in a few years.’ [B2} | AG |
| Theme 5: Organizational structure | | |  |
|  | Subtheme: Insurance/finances | |  |
|  |  | ‘Ah I think in terms of insurance. When, I saw that in Germany, for example, there are also people who, after three weeks, for example, they still couldn't bend their knee enough to actually be able to go home. And what do they do, they just book an extra week.’ [B4] | AG |
|  |  | ‘But we had to pay for that physio ourselves (in the Netherlands). Because you get up to so many treatments. So, and therefore I also say, the insurances, you do have to know well what kind of treatments you need, how many treatments you need, and yeah, if it stops at ten, or at twelve, whatever, and you are still not there, you are not ready, then you have to pay them by yourself.’ [B2P] | DN |
|  |  | ‘Well, here in the Netherlands it is like this, if you have had your ten appointments with the physiotherapist, and your insurance says, number eleven you have to pay yourself and those after as well, than you think, well whatever then, then I will walk worse for a while.’ [B4] | DN |
|  | Subtheme: Planning and information | |  |
|  |  | ‘Yes, because we really had to go after it for him when we came from Germany. That was kind of annoying, because then he's already in the loop to get physiotherapy. And we had to figure out which physiotherapist ourselves. And now? And then another two-month wait, you get on the waiting list, you get this.’ [B2P] | DN |
|  |  | ‘Well for the first two days, then I would have wished that they had given me some guidance on how or what.’ [B5] | O |
|  |  | ‘But just for the first day I would find it very nice, if I would have to go somewhere again, to have some guidance on how or what. And then I’ll manage. Where is everything, where do I have to go, where is the kitchen, where is this or whatever.’ [B5] | O |
|  |  | ‘I also think that that difference between / what I understood as well is that the people who are operated on in Germany are still in hospital for ten days and then they go to the rehabilitation center, but we went there after three four days. I think yes that that difference will have to be leveled out. [B3] | O |
|  | Subtheme: Communication | |  |
|  |  | ‘But I was also unlucky, that my papers were not there (in the rehabilitation center). (...) So, then everything had to be done manually, and ask and ask and ask and ask. Yes where are those papers? Yes, how should I know.. Yes where are those papers? I don't know. So those did not arrive on time, and the second day after that they arrived.’ [B5] | O |
|  |  | ‘It was a process indeed no, how you drop him, and you hear nothing for a while,, I got no communication nothing. And at one point he needs for example needed swimming shorts, and I was like, how do I do that, should I drive there, but then again that was not allowed, so I um, yes we went after the postal address and such. Then I posted it.’ [B2P] | O |
|  |  | ‘Well I also had a drawback. Because I was finished there in Bad-Zwischenahn, so I had to go to physiotherapy here. But they still needed the letter from Germany first. And they needed the letter from orthopedics before they would start treating me. I waited two weeks for that, and found that quite, quite unfortunate. So I would say before the operation, start with physiotherapy, so that you can already be scheduled after the operation, because I thought it was a waste of time, and you could feel your muscle strength diminishing.’ [B1] | O |

AN, Advantages The Netherlands; AG, Advantages Germany; DN, Disadvantages The Netherlands’; DG, Disadvantages Germany; O, Other
